# The Influence of Ghrelin on the Development of Dextran Sodium Sulfate-Induced Colitis in Rats

**DOI:** 10.1155/2015/718314

**Published:** 2015-12-02

**Authors:** Aleksandra Matuszyk, Dagmara Ceranowicz, Zygmunt Warzecha, Piotr Ceranowicz, Krzysztof Fyderek, Krystyna Gałązka, Jakub Cieszkowski, Joanna Bonior, Jolanta Jaworek, Małgorzata Pihut, Artur Dembiński

**Affiliations:** ^1^Department of Physiology, Faculty of Medicine, Jagiellonian University Medical College, 16 Grzegórzecka Street, 31-531 Cracow, Poland; ^2^Department of Anatomy, Faculty of Medicine, Jagiellonian University Medical College, 12 Kopernika Street, 31-034 Cracow, Poland; ^3^Department of Pediatrics, Gastroenterology and Nutrition, Children's Hospital, Faculty of Medicine, Jagiellonian University Medical College, 256 Wielicka Street, 30-663 Cracow, Poland; ^4^Department of Pathology, Faculty of Medicine, Jagiellonian University Medical College, 16 Grzegórzecka Street, 31-531 Cracow, Poland; ^5^Department of Medical Physiology, Faculty of Health Sciences, Jagiellonian University Medical College, 12 Michałowskiego Street, 31-126 Cracow, Poland; ^6^Department of Dental Prosthetics, Institute of Dentistry, Faculty of Medicine, Jagiellonian University Medical College, 4 Montelupich Street, 31-155 Cracow, Poland

## Abstract

Ghrelin has protective and therapeutic effects in the gut. The aim of present studies was to investigate the effect of treatment with ghrelin on the development of colitis evoked by dextran sodium sulfate (DSS).* Methods.* Studies have been performed on rats. Colitis was induced by adding 5% DSS to the drinking water for 5 days. During this period animals were treated intraperitoneally twice a day with saline or ghrelin given at the dose of 8 nmol/kg/dose. On the sixth day, animals were anesthetized and the severity of colitis was assessed.* Results.* Treatment with ghrelin during administration of DSS reduced the development of colitis. Morphological features of colonic mucosa exhibited a reduction in the area and deep of mucosal damage. Ghrelin reversed the colitis-induced decrease in blood flow, DNA synthesis, and superoxide dismutase activity in colonic mucosa. These effects were accompanied by a decrease in the colitis-evoked increase in mucosal concentration of interleukin-1*β* and malondialdehyde. Treatment with ghrelin reversed the DSS-induced reduction in body weight gain.* Conclusions.* Administration of ghrelin exhibits the preventive effect against the development of DSS-induced colitis. This effect seems to be related to ghrelin's anti-inflammatory and antioxidative properties.

## 1. Introduction

Crohn's disease and ulcerative colitis are the two main forms of inflammatory bowel disease (IBD) [1]. It is currently suggested that IBD results from an abnormal immunological response to micro flora present in the digestive system and its pathogenesis is complex and requires coexistence of factors of environmental and genetic nature [1–3]. There are numerous methods to treat IBD, but medication that would ensure permanent therapeutic effect has not been found so far [1, 3]. Therefore, it is vital to search for the new therapeutic strategies.

Ghrelin was primary discovered in the human and rat stomach by Kojima et al. [4]. The stomach is a major source of circulating ghrelin [5], but it was also found in other organs such as the bowel, pancreas, kidney, pituitary gland, and hypothalamus [6–8]. Ghrelin acts on a receptor primarily known as the growth hormone secretagogue receptor (GHS-R), which was currently renamed the ghrelin receptor (GRLN-R) [9]. GRLN-R is mainly present in the pituitary gland and hypothalamus, but it was also in other tissues of the body [6, 9]. Ghrelin stimulates growth hormone secretion [4] and body weight gain by an increase in food intake and a decrease in utilization of fat tissue [9, 10]. Besides these effects, ghrelin demonstrates anti-inflammatory and therapeutic properties in different organs of the body. GRLN-Rs are present, among others, in the immunological system cells, suggesting that ghrelin may modulate inflammatory response [11, 12].

In the gastrointestinal tract, administration of ghrelin reduces gastric injury [13–15] and accelerates the healing of gastric [16, 17], duodenal [16, 18], and oral ulcers [19] evoked by different damaging agents. Pretreatment with ghrelin inhibits the development of experimental acute pancreatitis [20, 21] and its administration accelerates recovery in this disease [22–24].

The role of ghrelin in IBD is not clear. Clinical studies have shown that expression of mRNA for ghrelin is significantly upregulated in colonic mucosa in patients with IBD and this effect is well-correlated with a grade of inflammation [25, 26].

There are animal experimental studies concerning the role of ghrelin in the development and healing of colitis. Some studies have shown that administration of exogenous ghrelin accelerates the healing of colitis evoked by trinitrobenzene sulfonic acid (TNBS) in rats [25] and mice [27]. Therapeutic effect of ghrelin has been also found in the course of dextran sodium sulfate- (DSS-) induced colitis in rats [28]. In contrast to those data are results obtained by De Smet et al. [29]. They have reported that endogenous and exogenous ghrelin enhance the colonic manifestation of DSS-induced colitis in mice. Proinflammatory properties of ghrelin in the colon have been also postulated by Zhao et al. [30]. They have found that ghrelin stimulates interleukin-8 gene expression through protein kinase C-mediated NF-kappaB pathway in human colonic epithelial cells.

The objective of the present study was to determine the influence of treatment with ghrelin on the development of DSS-induced colitis in rats.

## 2. Materials and Methods

### 2.1. Animals and Treatment

The research was performed on forty Wistar male rats weighing 180–250 g and conducted following the experimental protocol approved by the First Local Committee of Ethics for the Care and Use of Laboratory Animals in Cracow. During the study, animals had free access to food and fluids.

Animals were randomly divided into four groups: (1) control rats without colitis treated intraperitoneally (i.p.) with saline; (2) rats without colitis treated i.p. with ghrelin; (3) rats treated i.p. with saline during induction of colitis; (4) rats treated i.p. with ghrelin during induction of colitis. Each experimental group consisted of 10 animals.

Colitis was induced by adding 5% DSS (40 kDa, Sigma-Aldrich, St. Louis, USA) to the drinking water for 5 days (groups 3 and 4).

Other animals had free access to tap water (groups 1 and 2). During this 5-day period, animals were treated i.p. twice a day with saline (groups 1 and 3) or ghrelin (groups 2 and 4). Rat ghrelin (Yanaihara Institute, Shizuoka, Japan) was given at the dose of 8 nmol/kg/dose. This dose was chosen because previous studies have shown that ghrelin given at the dose 8 nmol/kg/dose exhibits strong and repeatable therapeutic effect in the healing of gastric, duodenal, and oral ulcers [16–19].

On the sixth day, rats were anesthetized with ketamine (50 mg/kg i.p., Bioketan, Vetoquinol Biowet, Gorzów Wielkopolski, Poland) and the severity of colitis was assessed.

### 2.2. Measurement of the Body Weight Gain and Daily Water Intake

In the study the effect of ghrelin and DSS on the water intake and weight gain of the animals was identified. Body weight of each animal was checked at the beginning and after each day of study. Daily water intake was defined as the difference between the initial volume of water and the volume remaining in the water container before refilling.

### 2.3. Measurement of Colonic Blood Flood and Mucosal Lesions

Six days after induction of colitis, rats were anesthetized with ketamine. After opening the abdominal cavity and exposing the colon, the measurement of colonic blood flow volume was performed using laser Doppler flowmeter (PeriFlux 4001 Master Monitor, Perimed AB, Järfälla, Sweden), in accordance with the methodology described before [31]. After measurement of colonic blood flow, the area of mucosal damage was measured, using a computerized planimeter (Morphomat, Carl Zeiss, Berlin, Germany) in accordance with the method described earlier [31].

### 2.4. Biochemical Analysis

After measurement of colonic blood flow and lesions area, biopsy samples of mucosa from the colon were taken for determination of mucosal DNA synthesis (an index of mucosal cell proliferation), concentration of proinflammatory interleukin-1*β*, concentration of malondialdehyde (as an index of lipid peroxidation), and activity of superoxide dismutase (SOD) (one of the endogenous mechanisms for elimination of free oxygen radicals).

### 2.5. Determination of DNA Synthesis in Colonic Mucosa

DNA synthesis was determined by measurement of [^3^H]thymidine incorporation ([6-^3^H]- thymidine, 20–30 Ci/mmol, Institute for Research, Production and Application of Radioisotopes, Prague, Czech Republic) into mucosal DNA as described previously [32]. The incorporation of labeled thymidine into DNA was determined by counting 0.5 mL DNA-containing supernatant in a liquid scintillation system. The rate of DNA synthesis was expressed as tritium disintegrations per minute per *μ*g of DNA (dpm/*μ*g DNA).

### 2.6. Determination of Interleukin-1*β* Concentration in Colonic Mucosa

Samples of colonic mucosa were homogenized in ice-cold phosphate-buffered saline (PBS, 20 mM, pH 7.4). Homogenate was centrifuged at 1,500 g for 10 min at 4°C. Content of interleukin-1*β* in the supernatant was measured using the BioSource Cytoscreen rat IL-1*β* kit (BioSource International, Camarillo, California, USA) based on ELISA. Concentration of interleukin-1*β* in duodenal mucosa was expressed as ng per g of tissue.

### 2.7. Determination of Malondialdehyde Concentration in Colonic Mucosa

Peroxidation of lipids in colonic mucosa was tested by measurement of malondialdehyde (MDA) concentration using commercial kit Bioxytech LPO-586 (OxisResearch, OXIS Health Products, Inc., Portland, OR, USA), as described previously [33]. Mucosa was homogenized in ice-cold Tris buffer (20 mM, pH 7.4) and centrifuged (3000 g at 4°C for 10 min) and supernatant was used for the assay.

### 2.8. Determination of Superoxide Dismutase Activity in Colonic Mucosa

To determine the activity of superoxide dismutase (SOD) in colonic mucosa, tissue was homogenized in 20 mM HEPES buffer, pH 7.2, containing 1 mM EGTA, 210 mM mannitol, and sucrose. Homogenate was centrifuged at 1,500 g for five min at 4°C. Activity of SOD in the supernatant was measured using Superoxide Dismutase Assay Kit (Cayman Chemical Company, Ann Arbor, MI, USA). Results have been expressed in units per g of colonic mucosa.

### 2.9. Histological Examination of the Colon

Samples of the colon were fixed in 10% buffered formaldehyde and embedded in paraffin. Paraffin sections were stained with hematoxylin and eosin and examined by the pathologist uninformed about treatment given. The histological grading of colonic damage such as ulceration, inflammation, depth of the lesion, and fibrosis was determined using a scale previously presented by Vilaseca et al. [34].

### 2.10. Statistical Analysis

Results were presented as a mean value ± standard error (SEM). Statistical assessment was done through one-way analysis of variance followed by Tukey's multiple comparison test using GraphPad Prism (GraphPad Software, San Diego, CA, USA). Differences were considered to be statistically significant if *P* was less than 0.05.

## 3. Results

**Figure 1:**
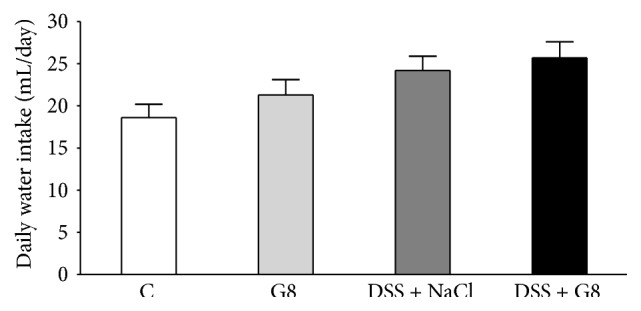
Influence of dextran sodium sulfate (DSS) administered in drinking water and saline (NaCl) or ghrelin given intraperitoneally at the dose of 8 nmol/kg/dose (G8) on daily water intake. Mean ± SEM. *N* = 10 animals in each group.

In control group of animals, mean daily water intake was 18.6 ± 1.6 mL (Figure 1). Intraperitoneal administration of ghrelin at the dose of 8 nmol/kg/dose resulted in a slight increase in water intake in animals watered with tap water, but this effect was statistically insignificant. Also, administration of DSS in drinking water was without a significant effect on the daily intake of water. In the group of animals receiving in drinking water 5% solution of DSS and treated with ghrelin, daily intake of water reached was the greatest value, but also this effect was not statistically significant (Figure 1).

**Figure 2:**
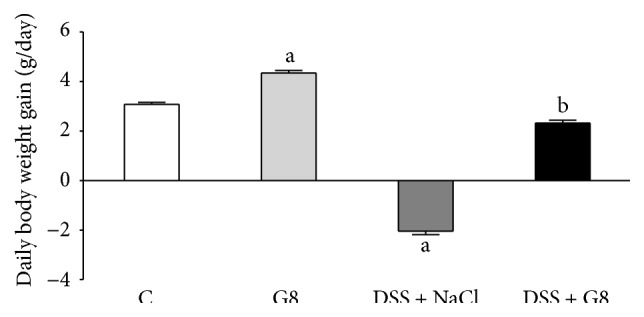
Influence of dextran sodium sulfate (DSS) administered in drinking water and saline (NaCl) or ghrelin given intraperitoneally at the dose of 8 nmol/kg/dose (G8) on daily body weight gain. Mean ± SEM. *N* = 10 animals in each group. ^a^ *P* < 0.05 compared to control (C); ^b^ *P* < 0.05 compared to DSS plus NaCl.

In control animals receiving tap water and treated intraperitoneally with saline, a mean daily body weight gain within five days of observation was 3.1 ± 0.1 g (Figure 2). Administration of ghrelin led to statistically significant increase by 41% in that parameter. Addition of DSS to drinking water resulted in a decrease in body weight in comparison to initial body weight. Administration of ghrelin significantly reversed the body weight loss in animals watered with 5% solution of DSS, leading to a body weight gain similar to that observed in the control animals (Figure 2).

**Figure 3:**
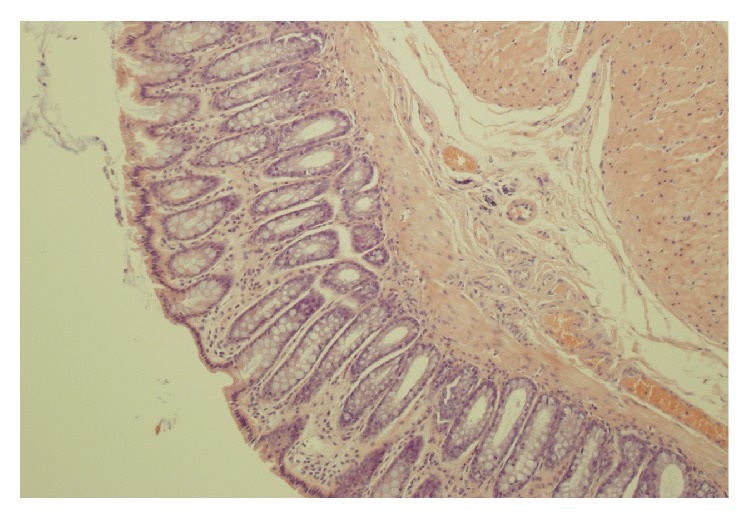
A typical microscopic image of colonic mucosa taken from the control animals watered with tap water and treated intraperitoneally with saline (NaCl). Hematoxylin-eosin counterstain. Original magnification 200x.

**Figure 4:**
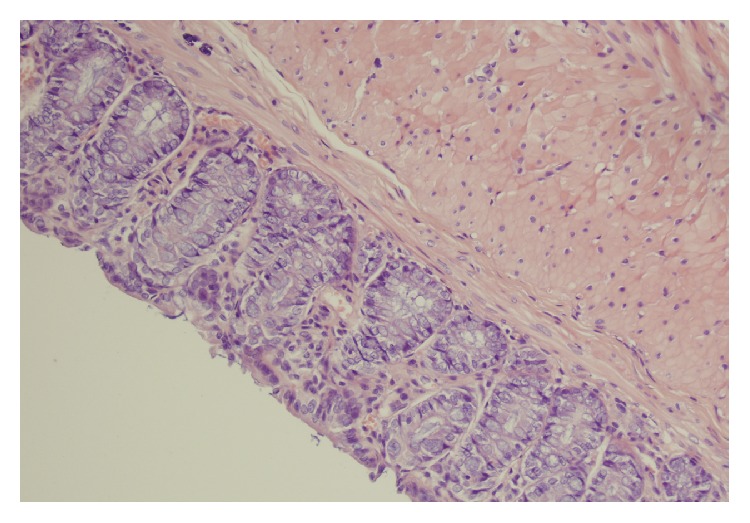
A typical microscopic image of colonic mucosa taken from the animals watered with tap water and treated intraperitoneally with ghrelin. Hematoxylin-eosin counterstain. Original magnification 400x.

**Figure 5:**
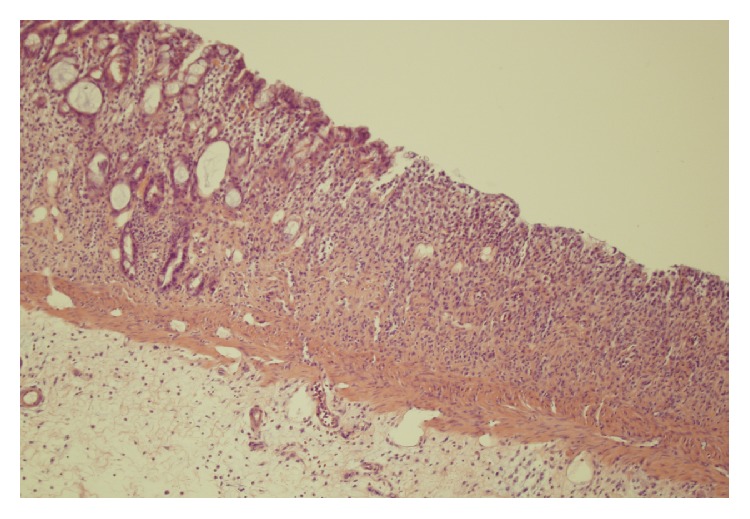
A typical microscopic image of colonic mucosa taken from the animals watered with DSS solution and treated intraperitoneally with saline. Hematoxylin-eosin counterstain. Original magnification 100x.

**Figure 6:**
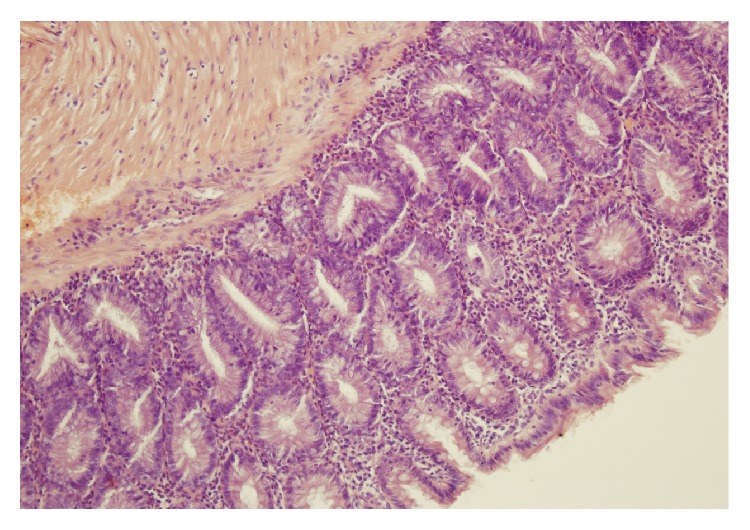
A typical microscopic image of colonic mucosa taken from the animals watered with DSS solution and treated intraperitoneally with ghrelin. Hematoxylin-eosin counterstain. Original magnification 200x.

No macroscopic damage of the colon was observed in all rats used in the present study. Also, no damage was observed in microscopic morphological features of the colon obtained from control rats or rats treated with ghrelin and watered with tap water (Table 1, Figures 3 and 4). In the case of animals watered with aqueous solution of DSS and treated with saline, microscopic examination revealed the presence of small or large erosion extending into the submucosa or* muscularis propria*. Also moderate or severe inflammatory infiltrations and mild fibrosis were observed (Table 1, Figure 5). In most cases of animals watered with aqueous solution of DSS, administration of ghrelin prevented the formation of colonic lesions but failed to affect inflammatory infiltration (Table 1, Figure 6).

**Figure 7:**
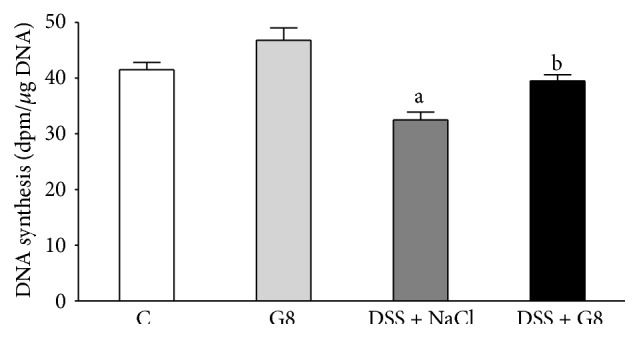
Influence of dextran sodium sulfate (DSS) administered in drinking water and saline (NaCl) or ghrelin given intraperitoneally at the dose of 8 nmol/kg/dose (G8) on DNA synthesis in colonic mucosa. Mean ± SEM. *N* = 10 animals in each group. ^a^ *P* < 0.05 compared to control (C); ^b^ *P* < 0.05 compared to DSS plus NaCl.

In the animals from the control group, the rate of DNA synthesis in colonic mucosa reached a value of 41.5 ± 1.3 dpm/*μ*g DNA (Figure 7). In animals watered with tap water, administration of ghrelin resulted in an increase in mucosal DNA synthesis, but this effect was statistically insignificant. Watering with DSS significantly reduced DNA synthesis by 22% in comparison to a level observed in control animals. Administration of ghrelin partly but significantly reversed the DSS-evoked reduction in DNA synthesis in colonic mucosa (Figure 7).

**Figure 8:**
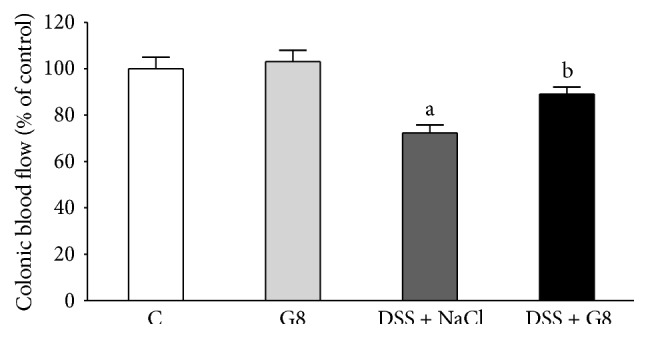
Influence of dextran sodium sulfate (DSS) administered in drinking water and saline (NaCl) or ghrelin given intraperitoneally at the dose of 8 nmol/kg/dose (G8) on mucosal blood flow in the colon. Mean ± SEM. *N* = 10 animals in each group. ^a^ *P* < 0.05 compared to control (C); ^b^ *P* < 0.05 compared to DSS plus NaCl.

Administration of ghrelin failed to affect mucosal blood flow in the colon in animals watered with tap water (Figure 8). Induction of colitis by administration of DSS led to a statistically significant decrease in colonic mucosal blood flow by about 28%, when compared to a value observed in animals from control group (Figure 8). Administration of ghrelin partly, but significantly, reversed the DSS-evoked decrease in mucosal blood flow in the colon.

**Figure 9:**
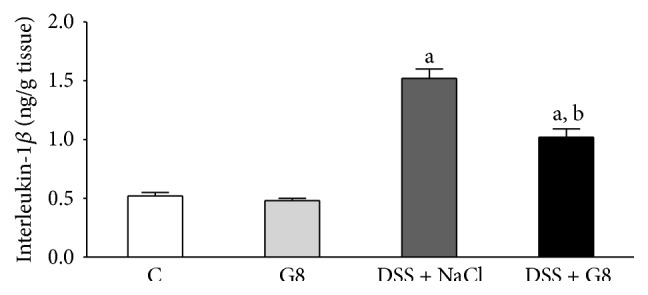
Influence of dextran sodium sulfate (DSS) administered in drinking water and saline (NaCl) or ghrelin given intraperitoneally at the dose of 8 nmol/kg/dose (G8) on interleukin-1*β* concentration in colonic mucosa. Mean ± SEM. *N* = 10 animals in each group. ^a^ *P* < 0.05 compared to control (C); ^b^ *P* < 0.05 compared to DSS plus NaCl.

The concentration of proinflammatory interleukin-1*β* (IL-1*β*) in colonic mucosa in control animals was 0.50 ± 0.03 ng/g of tissue (Figure 9). Administration of ghrelin was without significant effect on IL-1*β* concentration in colonic mucosa in animals watered with tap water. Induction of colitis by watering with DSS solution led to a 3-fold increase in IL-1*β* concentration in colonic mucosa. Administration of ghrelin partly, but significantly, reversed the DSS-evoked increase in that parameter (Figure 9).

**Figure 10:**
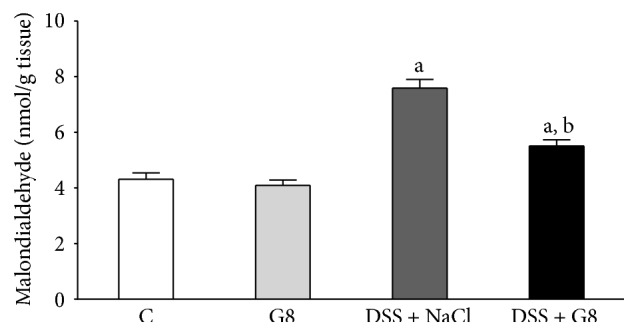
Influence of dextran sodium sulfate (DSS) administered in drinking water and saline (NaCl) or ghrelin given intraperitoneally at the dose of 8 nmol/kg/dose (G8) on malondialdehyde concentration in colonic mucosa Mean ± SEM. *N* = 10 animals in each group. ^a^ *P* < 0.05 compared to control (C); ^b^ *P* < 0.05 compared to DSS plus NaCl.

Administration of ghrelin was without significant effect on the level of malondialdehyde (MDA) in colonic mucosa in animals watered with tap water (Figure 10). Addition of DSS to water resulted in almost a 2-fold increase in the concentration of MDA in the colonic mucosa in comparison to concentration observed in the control animals. In rats watered with DSS solution, administration of ghrelin resulted in a statistically significant reduction in the concentration of MDA in the colonic mucosa (Figure 10).

**Figure 11:**
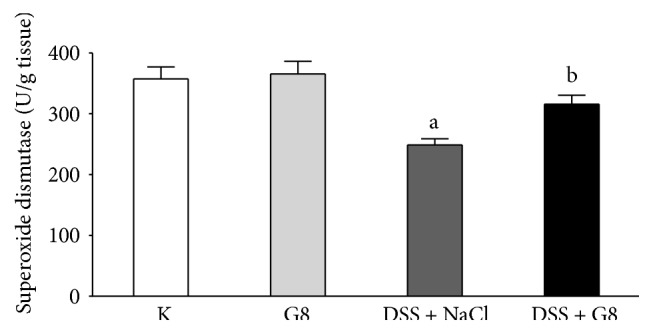
Influence of dextran sodium sulfate (DSS) administered in drinking water and saline (NaCl) or ghrelin given intraperitoneally at the dose of 8 nmol/kg/dose (G8) on superoxide dismutase activity in colonic mucosa. Mean ± SEM. *N* = 10 animals in each group. ^a^ *P* < 0.05 compared to control (C); ^b^ *P* < 0.05 compared to DSS plus NaCl.

In control saline-treated animals, superoxide dismutase (SOD) activity in colonic mucosa was low and reached a value of 357.2 ± 19.8 U/g of tissue (Figure 11). Administration of ghrelin failed to affect SOD activity in colonic mucosa in rats without induction of colitis. Induction of colitis by DSS solution resulted in a significant decrease in SOD activity in colonic mucosa. In those rats, administration of ghrelin partly, but significantly, reversed the DSS-evoked decrease in mucosal SOD activity in the colon (Figure 11).

## 4. Discussion

Our present study has provided several important observations regarding the influence of exogenous ghrelin on the integrity of the colonic mucosa. We have found that treatment with ghrelin exhibits protective effect on the colon. Ghrelin given during administration of DSS inhibited the development of DSS-induced colitis. This effect was manifested as an improvement of colonic morphology in the microscopic examination, a partial reversion of the colitis-evoked reduction in mucosal cell vitality and an increase in mucosal blood flow, as well as a decrease in biochemical indices of inflammation.

DNA synthesis, measured by incorporation of labeled thymidine into DNA, is an index of cell vitality and cell proliferation [32]. Our present study has shown that induction of colitis is associated with a decrease in mucosal DNA synthesis in the colon. The rate of mucosal DNA synthesis was inversely proportional to the grade of colonic damage. In rats without induction of colitis, administration of ghrelin was without a significant influence on DNA synthesis in colonic mucosa. On the other hand, administration of ghrelin in animals watered with DSS solution caused a considerable reversal of DSS-evoked decrease in DNA synthesis in the colon. These findings indicate that protective effect of ghrelin in the colon is related, at least in part, to an increase in vitality of cells in colonic mucosa.

The maintenance of normal blood flow through microcirculation plays a fundamental role in the protection and healing of mucosa in the gut [35–38]. Previous studies have shown that exposure of gastric mucosa to potentially damaging factors results in little or no damage, as long as an adequate blood flow is sustained. On the other hand, a decrease in mucosal blood flow increases the severity and extension of gastric lesions [35]. The importance of appropriate blood flow in maintaining mucosal integrity and healing of mucosa damage has been also found in other parts of the digestive tract system such as the oral cavity [19], esophagus [36], duodenum [37], and colon [38]. Findings of our present study are in harmony with those data. Administration of ghrelin during colitis induction by DSS led to a significant restitution of appropriate blood flood through colonic microcirculation. This observation indicates that ghrelin's protective effect on colonic mucosa is connected with its influence on mucosal blood flow. A question remains, however, whether the ghrelin-evoked improvement of blood flood through colonic mucosa in rats with colitis is a mechanism or a result of ghrelin's protective effect in the colon.

A next interesting discovery of our present study concerned the influence of DSS and ghrelin administration on biochemical indices of inflammation and oxidative stress. Interleukin-1*β* (IL-1*β*) plays a crucial role in the induction of local inflammation and systemic acute phase response. Also, it is responsible for the release of subsequent proinflammatory cytokines in the biochemical cascade of inflammation [39, 40]. Our present study has shown that induction of colitis by DSS leads to a damage and inflammatory infiltration of colonic mucosa and increases mucosal concentration of IL-1*β*, whereas administration of ghrelin decreases mucosal concentration of IL-1*β* in rats watered with DSS solution. These data indicate that ghrelin reduces a local inflammatory response in colitis induced by DSS and this effect seems to be one of the mechanisms involved in the ghrelin-evoked protective effect in the colon.

Tissue concentration of malondialdehyde (MDA) is a biological marker of oxidative stress [41], whereas superoxide dismutase (SOD), catalase, and glutathione peroxidase are the main enzymes responsible for reactive oxygen species (ROS) neutralization [42]. Involvement of ROS has been shown, among others, in the pathogenesis of gastric [42, 43] and colonic damage [44]. In our present study, administration of ghrelin failed to affect MDA concentration or SOD activity in colonic mucosa in rats watered with tap water. Induction of colitis increased the concentration of MDA, a lipid peroxidation product, in colonic mucosa and this effect was associated with a reduction in mucosal SOD activity, leading to additional disruption of redox balance. On the other hand, administration of ghrelin partly, but significantly, reversed the DSS-induced increase in mucosal MDA concentration and this effect was associated with partial restoration of mucosal activity of SOD. These observations indicate that treatment with ghrelin reduces the colitis-induced oxidative stress in colonic mucosa and improves the antioxidant defense in this inflammation.

Ghrelin stimulates growth hormone and IGF-1 secretion and as promitotic agent can be potentially directly or indirectly involved in carcinogenesis [45, 46]. The stomach is a main source of circulating ghrelin, but this peptide is also widely expressed in other tissues in normal and malignant conditions [6–8, 46]. Several endocrine and nonendocrine tumors, such as pituitary adenomas, thyroid tumors, carcinoids, lung and breast cancer, and colorectal cancer as well as gastric and pancreatic adenocarcinoma express ghrelin and ghrelin's receptor at both mRNA and protein level [46]. However, the influence of ghrelin on cancer risk is unclear. There are some early studies showing that ghrelin increases proliferation and invasiveness of pancreatic adenocarcinoma [47], hepatoma [48], and adrenocortical tumor cells [49]. On the other hand, numerous recent clinical and experimental studies [8, 50, 51], including our present data, indicate that ghrelin exhibits anti-inflammatory effect and plays a role in protecting against cancer-related inflammation [52] and inflammation-associated carcinogenesis [53]. For this reason, clinical observations also indicate that low levels of ghrelin are associated with an increased risk of esophageal, gastric, and esophagogastric junctional adenocarcinoma [54, 55]. Similar antitumor effect of ghrelin has been detected in breast cancer. Patients with breast cancer expressing ghrelin have a 2.5–3-fold lower risk for recurrence or breast cancer death than those lacking ghrelin expression [56]. Moreover, recent clinical studies indicate that ghrelin can be useful in the postoperative support and treatment of anorexia-cachexia syndrome of cancer patients [57, 58]. Treatment with ghrelin for cancer-related cachexia improves the patients' general conditions and their quality of life [52, 57, 58].

Finally, we conclude that administration of ghrelin protects the large bowel against the development of DSS-induced colitis. This observation suggests that treatment with ghrelin can be useful in maintaining the IBD patients in remission.

## Figures and Tables

**Table 1:** Influence of dextran sodium sulfate (DSS) administered in drinking water and saline (NaCl) or ghrelin given intraperitoneally at the dose of 8 nmol/kg/dose on morphological signs of colonic damage.

	Morphological changes			
	Size of lesions	Inflammatory infiltration	Depth of lesions	Fibrosis
	(0–2)	(0–3)	(0–3)	(0–3)
Control				
Scoring observed in individual animals	0, 0, 0, 0, 0	0, 0, 0, 0, 0	0, 0, 0, 0, 0	0, 0, 0, 0, 0
	1, 0, 0, 0, 0	0, 0, 0, 0, 0	1, 0, 0, 0, 0	0, 0, 0, 0, 0
The predominant grading	0	0	0	0

Ghrelin				
Scoring observed in individual animals	0, 0, 0, 0, 0	0, 0, 0, 0, 0	0, 0, 0, 0, 0	0, 0, 0, 0, 0
	0, 0, 0, 0, 0	0, 0, 0, 0, 0	0, 0, 0, 0, 0	0, 0, 0, 0, 0
The predominant grading	0	0	0	0

DSS				
Scoring observed in individual animals	1, 1, 2, 1, 2	2, 2, 3, 2, 3	1, 1, 2, 1, 1	0, 1, 2, 1, 1
	1, 2, 2, 2, 2	2, 3, 3, 2, 2	1, 1, 1, 1, 1	1, 1, 1, 1, 1
The predominant grading	1-2	2-3	1	1

DSS + Ghrelin				
Scoring observed in individual animals	0, 1, 0, 0, 0	2, 3, 2, 2, 3	0, 1, 0, 0, 0	0, 0, 0, 0, 0
	1, 0, 1, 0, 0	1, 2, 3, 2, 3	1, 0, 1, 0, 0	0, 0, 1, 0, 0
The predominant grading	0	2-3	0	0
